# Supplementary material for: The interactome of intact mitochondria by cross-linking mass spectrometry provides evidence for coexisting respiratory supercomplexes
Source: Mol Cell Proteomics. 2017 Dec 8;17(2):216–32. doi: 10.1074/mcp.RA117.000470 (PMC5795388; doi:10.1074/mcp.RA117.000470)
Supplement: Supplemental Data [file supp_RA117.000470_133922_0_supp_23978_fzffwf.zip › spectra_annotation/mito_spectra_annotation/251-1-6-1-7-1.pdf]

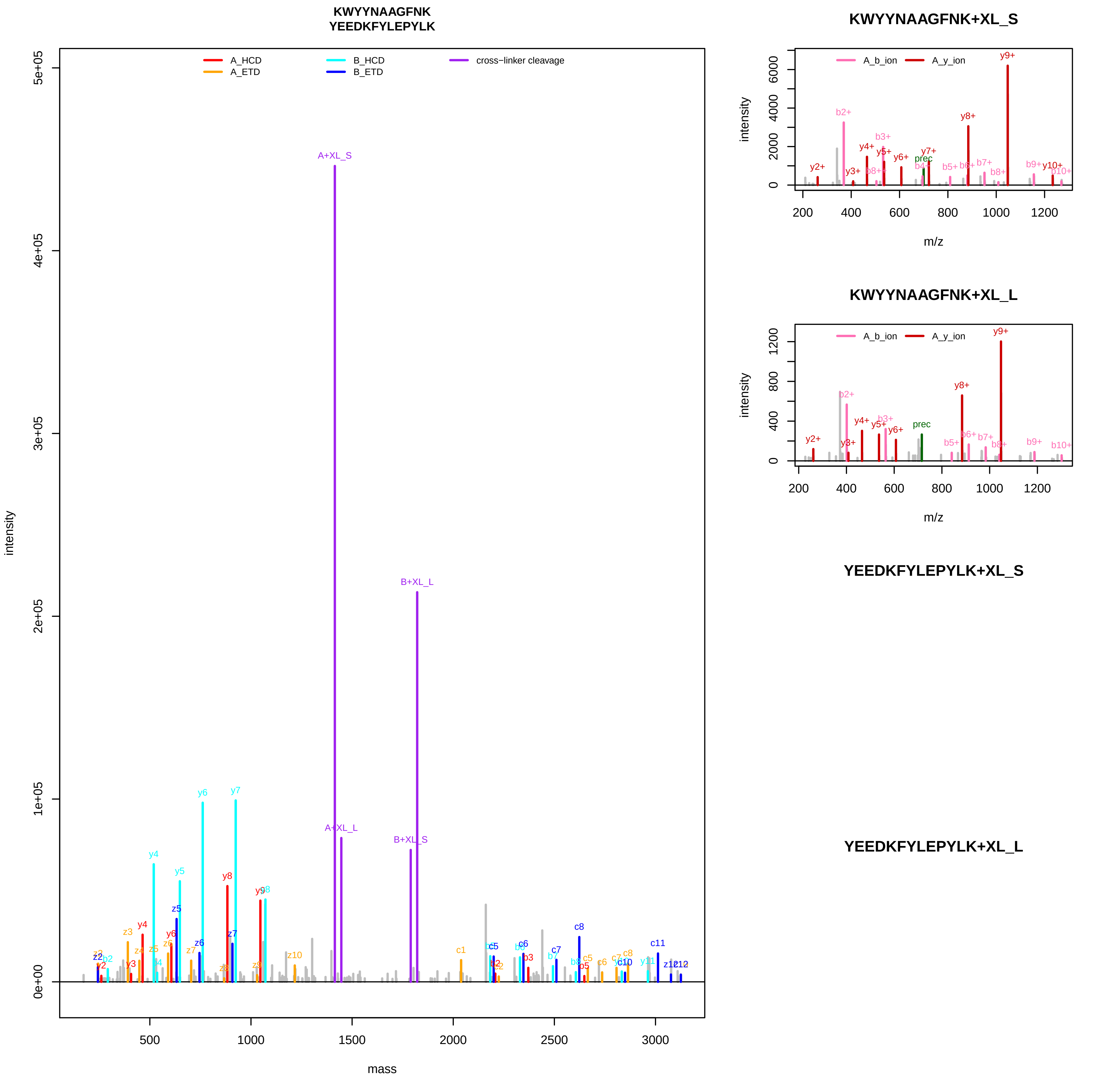
KWYYNAAGFNK
YEEDKFYLEPYLK
A_HCD
A_ETD
B_HCD
B_ETD
cross−linker cleavage
A+XL_S
B+XL_L
A+XL_L
B+XL_S
y4
y5
y6
y7
y8
y9
z3
z5
z6
z7
c1
c5
c6
c7
c8
c10
c11
y11
intensity
mass
0e+00
1e+05
2e+05
3e+05
4e+05
5e+05
500
1000
1500
2000
2500
3000
KWYYNAAGFNK+XL_S
A_b_ion
A_y_ion
y9+
y8+
b2+
b3+
y4+
y5+
y6+
y7+
prec
y2+
y3+
b5+
b6+
b7+
b8+
b9+
y10+
b10+
intensity
m/z
0
2000
4000
6000
200
400
600
800
1000
1200
KWYYNAAGFNK+XL_L
A_b_ion
A_y_ion
y9+
y8+
b2+
b3+
y4+
y5+
y6+
prec
y2+
y3+
b5+
b6+
b7+
b8+
b9+
b10+
intensity
m/z
0
400
800
1200
200
400
600
800
1000
1200
YEEDKFYLEPYLK+XL_S
YEEDKFYLEPYLK+XL_L
